# Pulmonary Vein Isolation with Different Single-Shot Ablation Devices in Patients with Atrial Fibrillation: A Prospective Observational Single-Center Clinical Evaluation

**DOI:** 10.3390/medicina62101828

**Published:** 2026-09-22

**Authors:** Wiktor Czarkowski, Bartłomiej Popielarz, Agata Kukfisz, Michał Skrzypek, Mariusz Gąsior, Krzysztof Myrda

**Affiliations:** 1 2nd Department of Cardiology and Angiology, Silesian Centre for Heart Diseases, 41-800 Zabrze, Poland; 2 Department of Biostatistics, Faculty of Public Health in Bytom, Medical University of Silesia, 40-055 Katowice, Poland; mskrzypek@sum.edu.pl; 3 3rd Department of Cardiology, Faculty of Medical Sciences in Zabrze, Medical University of Silesia, 40-055 Katowice, Poland

**Keywords:** atrial fibrillation, single-shot devices, ablation, pulmonary vein isolation

## Abstract

*Background and Objectives*: The conventional technique for atrial fibrillation (AF) ablation has gradually been supplemented by ‘single-shot’ devices. Comparisons of devices using three different energy sources are rare. We aimed to evaluate the outcomes of patients with AF who underwent pulmonary vein isolation (PVI) using three various single-shot ablation techniques. We also examined factors associated with arrhythmia recurrence during the 12-month follow-up. *Materials and Methods*: We prospectively analyzed single-center data from consecutive adult patients undergoing PVI with different single-shot devices between January and December 2024. For cryoablation, a second-generation cryoballoon (AFA, Medtronic, Minneapolis, MN, USA) was used; for radiofrequency balloon ablation, the RFB catheter (Heliostar, Biosense Webster, California, CA, USA) was applied; and for the PFA-guided procedure, the Farawave (Boston Scientific, Massachusetts, MA, USA) ablation catheter was used. The procedural endpoint was to achieve intraoperative PVI. Arrhythmia recurrence was defined as any episode lasting >30 s documented by electrocardiographic recordings. *Results:* Ultimately, the analysis included 115 patients. Of these, 41 underwent PVI by cryoablation, 22 underwent RFB therapy, and 52 underwent PFA. The total skin-to-skin procedure time was the shortest among PFA-patients, primarily due to shorter left atrial dwell time. After 12 months of follow-up, 30 patients experienced recurrence of atrial arrhythmia. A larger left atrium diameter increased the risk of arrhythmia recurrence, whereas propafenone therapy at discharge was associated with a lower risk of recurrence during the 12-month follow-up. *Conclusions*: In this single-center observational study, PVI performed with PFA was associated with the shortest procedure time compared with procedures using CBA or RFB, with no difference in the risk of arrhythmia recurrence among the single-shot ablation modalities during the 12-month follow-up.

## 1. Introduction

Over the past decade, percutaneous ablation has become a primary treatment option for patients with atrial fibrillation (AF). Its benefits are highlighted in guidelines, which increasingly recommend catheter ablation [1,2,3]. The conventional ‘point-by-point’ radiofrequency (RF) ablation technique for pulmonary vein isolation (PVI) has gradually been supplemented by ‘single-shot’ devices. Among these, cryoablation has been the dominant technique, which, although comparable in efficacy, appeared to be technically simpler, less time-consuming, and associated with a significantly shorter learning curve [4]. The recently introduced new ablation energy source, pulsed field ablation (PFA), aims to reduce the risk of common complications associated with thermal energy devices [5].

Recent publications have focused on clinical experience with single-shot devices. Some studies have compared clinical outcomes with devices using different energy sources in randomized and nonrandomized designs. Comparisons among these three types of devices are rare [6,7,8,9,10]. Such comparisons primarily provide insight into the practical usefulness of these devices in the daily practice of electrophysiology laboratories. Despite some limitations, the use of a single-center laboratory model may allow conclusions to be drawn regarding the practical usefulness of the compared ablation energy sources during PVI procedures.

Using a wide range of devices available at our center, including single-shot ablation devices such as cryoballoon ablation (CBA), radiofrequency balloon (RFB), and a pulsed field ablation (PFA) system, we conducted a prospective, observational comparative analysis. The primary aim of this analysis was to evaluate the outcomes of patients with AF who underwent PVI using various single-shot ablation techniques, with particular emphasis on the risk of arrhythmia recurrence. Specifically, we analyzed selected parameters during hospitalization and after discharge. We also examined factors associated with arrhythmia recurrence during the 12-month postablation follow-up period.

## 2. Material and Methods

### 2.1. Study Population

We prospectively analyzed single-center data from consecutive adult patients undergoing ablation for symptomatic, antiarrhythmic drug-resistant AF between January and December 2024, all of whom underwent endocardial PVI with different single-shot devices. The following patients were excluded from the analysis: those with contraindications to ablation, including patients with thrombus in the cardiac chambers, including the left atrial appendage (LAA), before the procedure, and those who underwent atrial substrate modification using a different technique, such as surgical epicardial ablation. Before undergoing the procedure, all patients provided written informed consent for invasive diagnostic procedures and treatment. The flowchart of patients included in the final analysis is shown in Figure 1. This study was conducted in accordance with the Declaration of Helsinki as amended. The study was approved by the Bioethics Committee of the Medical University of Silesia (No. PCN/CBN/0022/KB1/136/23) and written informed consent was obtained from all patients.

### 2.2. Preablation Procedure

On admission, all patients underwent clinical and laboratory assessment, transthoracic echocardiography, and transesophageal echocardiography (TEE). In the absence of contraindications, antiarrhythmic drugs, including beta-blockers, propafenone, sotalol, and amiodarone, were continued during the hospital stay and at discharge. In-hospital anticoagulant treatment, including vitamin K antagonists (VKAs), such as warfarin, acenocoumarol, or direct oral anticoagulant drugs (DOACs), including dabigatran, rivaroxaban, or apixaban, was administered in accordance with current guidelines. When patients presented with an elevated international normalized ratio (INR) on admission, ablation was postponed until the next day(s).

### 2.3. Ablation Procedure

After obtaining written informed consent, the procedures were performed, and patients were under anesthetic care throughout the whole procedure. The decision regarding the choice of anesthesia was left to the discretion of the attending anesthesiologist. Routinely, two diagnostic catheters, i.e., a non-steerable catheter positioned at the right ventricular apex and a steerable catheter positioned in the coronary sinus, were inserted via femoral venous access using the Seldinger technique. Under fluoroscopic guidance, a single transseptal puncture was performed at the typical site, namely the fossa ovalis, using a non-steerable transseptal puncture system and a Brockenbrough needle. After the transseptal puncture, unfractionated heparin was titrated to achieve and maintain an activated clotting time between 300 and 350 s. Using an over-the-wire technique, a dedicated sheath was introduced for the selected device. Details of our ablation approach have been described previously [11]. PVI with different ablation energy sources was performed according to the established protocol [12,13,14,15]. The key factors influencing the choice of the therapeutic device were operator preferences and the availability of equipment in the electrophysiology laboratory. The procedural endpoint was to achieve intraoperative bidirectional conduction block in each of the pulmonary veins. For cryoablation, all patients underwent ablation using a 28-mm second-generation cryoballoon (Arctic Front Advance, Medtronic, Minneapolis, MN, USA). After exchange for the dedicated sheath, the cryoballoon was inserted and applications were delivered, with at least one application to each pulmonary vein [12]. Vessel occlusion was considered optimal when selective contrast injection showed no backflow into the left atrium (Figure 2A). Once occlusion was documented, cryoapplication was initiated. For radiofrequency balloon ablation, a dedicated introducer (Guidestar steerable sheath, Biosense Webster, California, CA, USA) and the RFB (Heliostar, Biosense Webster, California, CA, USA) were inserted into the left atrium (LA). A circular decapolar mapping catheter (Lassostar mapping catheter, Biosense Webster, California, CA, USA) was placed within its lumen and emerged from the tip of the RFB, permitting electroanatomic reconstruction of the LA. An initial antral circumferential RF application was then performed, with 15 W of energy delivered from each of the 10 electrodes: for 20 s from the three electrodes in contact with the posterior portion of the PV and for 60 s from the remaining seven electrodes (Figure 2B). The Faradrive (Boston Scientific, Massachusetts, MA, USA) sheath was advanced over a guidewire, and subsequently the Farawave (Boston Scientific, Massachusetts, MA, USA) ablation catheter was introduced into the LA over the guidewire. The PVs were treated in a clockwise direction, with energy applications delivered as a biphasic waveform unsynchronized with the cardiac rhythm, at an energy output of 2.0 kV, in a train of five consecutive pulses over a total ablation time of 2.5 s. Each vein was treated with eight energy applications, four in the flower configuration and four in the basket configuration (Figure 2C). Total skin-to-skin procedure time was measured from the moment groin puncture began until the procedure sheaths were removed. Fluoroscopy time and left atrial dwell time were measured from the moment the sheaths were inserted until the end of the procedure. The analyzed complications included transient ischemic attack (TIA), stroke, bleeding requiring blood transfusion, phrenic nerve palsy, pericardial tamponade, arteriovenous fistula at the puncture site, local hematoma managed conservatively, pseudoaneurysm at the puncture site, and pericardial effusion. The total complication rate was defined as the percentage of all analyzed complications, also in relation to the device used.

### 2.4. Postablation Follow up

Patients discharged after ablation remained under outpatient follow-up. Three follow-up visits were scheduled, each including Holter ECG monitoring. The first follow-up visit took place 3–6 months after ablation, the second at 6–9 months after ablation, and the third at 12 months after the procedure. Arrhythmia recurrence was defined as any sustained episode of AF, atrial flutter (AFl), or atrial tachycardia (AT) lasting >30 s and documented by electrocardiographic recording in patients presenting with subjective symptoms, or as an asymptomatic rhythm disturbance documented by Holter ECG monitoring or cardiac implantable electronic device (CIED) interrogation. The first 3 months after ablation were considered the blanking period. Management of arrhythmias during the blinded phase of the study was based on clinical symptoms, in accordance with current guidelines [3]. The decision to continue anticoagulation and antiarrhythmic therapy during follow-up was based on current clinical guidelines [3] and individualized risk reassessment. Based on the patient history obtained during subsequent outpatient follow-up visits and the available medical records, rehospitalization due to AF/AFl/AT or repeat ablation during the observation period was recorded.

### 2.5. Statistical Analysis

Quantitative variables are presented as the mean ± standard deviation or the median with the interquartile range (IQR). The significance of differences was assessed using the Kruskal–Wallis rank sum test, Wilcoxon rank sum test, Fisher’s exact test or Welch’s two- sample *t*-test. The risk of arrhythmia recurrence during the 12-month follow-up, depending on the device used, was compared using the log-rank test and presented with the Kaplan–Meier curve. A Cox regression model was used to identify factors associated with the risk of arrhythmia recurrence within 12 months after catheter ablation (CA). Baseline characteristics and data from the in-hospital period with a *p*-value of 0.3 or less were included in the univariate Cox analysis. Subsequently, a multivariable analysis, which included variables with a *p*-value of 0.1 or less in univariable analysis, was performed to identify independent predictors of arrhythmia recurrence. A *p*-value < 0.05 was considered statistically significant, and all analyses were performed using SAS software (version 9.4; SAS Institute, Inc., Cary, NC, USA) and R software version 4.4.3 (R Foundation for Statistical Computing, Vienna, Austria).

## 3. Results

### 3.1. Baseline Characteristics

Ultimately, the analysis included 115 patients, predominantly men, with a history of hypertension and paroxysmal AF. Of this cohort, 12 patients (10.4%) underwent the procedure as part of a repeat invasive treatment attempt. Of these, 41 underwent PVI by cryoablation, 22 underwent RFB therapy, and 52 underwent PFA. Patients undergoing PFA were significantly more likely to have previously undergone ablation therapy. Detailed data on the baseline characteristics of the study population are given in Table 1.

### 3.2. Perioperative and In-Hospital Data

Two-thirds of the procedures were performed under general anesthesia, including all patients undergoing PFA. The median total skin-to-skin procedure time was 75 min. This was the shortest among patients undergoing PFA and was primarily due to shorter left atrial dwell time. Neither fluoroscopy time nor exposure dose during subsequent procedures differed between groups. In-hospital complications occurred in 12 patients. Among these, pericardial effusion was significantly more common in patients treated with RFB. Detailed data on the perioperative parameters and the in-hospital course are given in Table 2.

### 3.3. Twelve-Month Follow-Up

All patients completed the follow-up. Thirty patients experienced ECG-documented recurrence of atrial arrhythmia (AF/AFl/AT) within 12 months after CA. The risk of atrial arrhythmia recurrence did not differ statistically between the groups according to the log-rank test. Data are presented as Kaplan–Meier curves (Figure 3). During the 12-month follow-up, the rate of hospitalization or repeat ablation due to recurrence of symptomatic arrhythmia was low, with no differences between the groups (Table 3).

### 3.4. Predictors of Atrial Arrhythmia Recurrence in 12-Month Follow-Up After Ablation

In univariable analysis, a higher risk of recurrence within 12 months after CA was observed in patients with persistent AF, chronic heart failure, higher creatinine levels at discharge, lower LVEF, larger LA diameter, longer procedure time and longer left atrial dwell time. Propafenone therapy at discharge significantly reduced the risk of atrial arrhythmia recurrence. The detailed data from the univariable analysis are presented in Table 4. In multivariable analysis, larger LA diameter was associated with a higher risk of recurrence at 12 months. Propafenone therapy at discharge significantly reduced the risk of arrhythmia recurrence. The detailed data from the multivariable analysis are presented in Table 5.

## 4. Discussion

To our knowledge, this observational study is the first prospective, single-center comparison of different single-shot devices for PVI using both PFA and thermal modalities, such as cryoablation and radiofrequency balloon ablation. The study included 115 patients, primarily with paroxysmal AF, the vast majority of whom underwent PVI for the first time.

The main findings from our single-center experience include a significant reduction in total skin-to-skin procedure time with PFA. This benefit was primarily due to a reduction in left atrial dwell time. Despite the observed differences, total fluoroscopy time remained comparable across all analyzed methods. Furthermore, PFA was associated with the lowest rate of analyzed intraprocedural complications, occurring in 3.8% of patients treated with PFA. Compared with PVI procedures using RFB and CBA, PFA was associated with the lowest rate of arrhythmia recurrence during the 12-month follow-up. In multivariable analysis, larger left atrial diameter increased the risk of arrhythmia recurrence during the 12-month follow-up, whereas the recommendation of propafenone at discharge decreased the risk of arrhythmia recurrence during the 12-month follow-up.

Currently, there are few studies in the literature evaluating selected parameters during PVI procedures using three single-shot devices simultaneously. In the only retrospective analysis conducted at an Austrian center, shorter procedure times were observed with PFA compared with CBA and RFB. In this analysis, the total procedure time with the PFA system was 55 min, which was significantly shorter than that of procedures performed with CBA and RFB (*p* < 0.001) [16]. The results of a meta-analysis comparing the use of PFA, CBA, and point-by-point RF ablation may also indicate a significant shortening of the PVI procedure using PFA [17]. This observation, however, may be inconsistent with reports in which the duration of the procedure, calculated in a manner analogous to that used in our study, did not differ significantly between PFA and RFB systems [18]. In another multicenter study using RFB and PFA systems, no differences were found in total left atrial dwell time [19]. However, the authors of this analysis did not specify whether electroanatomic mapping of the left atrium was performed during RFB procedures. This is important because this step was performed in every procedure we analyzed using this system, which increased the time during which the working device remained in the left atrium. However, no differences in procedure duration were noted in studies comparing RFB and CBA [6,8,20], which may suggest that factors influencing procedure duration include the operator’s experience with a given system, as well as the duration and number of applications [21].

Another aspect that should be considered when assessing the suitability of the three selected PVI devices is the occurrence of intraprocedural complications. In our study, 10% of patients experienced various intraprocedural complications. These included tamponade or pericardial effusion, most of which occurred during procedures using RFB. Their incidence was higher than that reported in the literature [6,8,20], which may be due to procedures performed by less experienced operators. The risk of air embolism remains significant when using single-shot devices [5,16]. In our study, this complication did not occur. However, special care should be taken when rinsing both the device itself and the working sheath, which has a large diameter (13–15 Fr).

Using available tools, such as regular outpatient visits, repeated ECG recordings, Holter monitoring, and access to records of urgent hospitalizations related to arrhythmia or the need for further ablation, we analyzed the long-term effectiveness of the procedure. Following PFA for atrial fibrillation, 86.5% of patients remained arrhythmia-free during the 12-month follow-up period. RF balloon ablation and CBA procedures were associated with a higher rate of arrhythmia recurrence, reaching 31.8% and 39%, respectively. Unfortunately, inconsistent results were obtained from statistical tests concerning this variable (Figure 3, Table 3). Although analysis using non-parametric tests revealed significant differences, the log-rank test showed no differences in the risk of arrhythmia recurrence. The Kruskal–Wallis rank sum test was used to compare variable values at a specific time point—12 months after the procedure. Despite the observation of statistically significant differences, it should be noted that this test does not account for censored data. Consequently, the log-rank test appears more reliable, as it considers the time to the event and fully accounts for censored data. The lack of statistical significance (*p* = 0.26) therefore indicates no difference in the risk of arrhythmia recurrence among the patients based on the therapeutic device used. In the previously cited comparison by Pavluk et al., the efficacy of rhythm control after PVI using RFB, CBA, and PFA was high, and arrhythmia recurrence was observed in only 6% to 9% of patients [16]. No statistical differences were observed, but the groups differed in the mean duration of follow-up. However, different conclusions can be drawn from comparisons of these modalities. Recent publications indicate the superiority of PFA over CBA [17,22]. Approximately 80% of patients undergoing both PFA and RFB remained arrhythmia-free after 12 months [18]. There are also data indicating no differences in this regard between RFB and CBA [20]. In the analysis by Vetta et al. comparing PFA and RFB, effectiveness in both groups was high, at 80% to 85%, and the differences were not statistically significant [19].

Finally, we would like to highlight the results of the multivariable analysis examining predictors of 12-month effectiveness. Factors influencing long-term effectiveness included left atrial size and propafenone therapy at discharge. However, the study group was small. Therefore, only a possible association can be inferred between the identified predictors and a reduced risk of arrhythmia recurrence. These limitations could have led to overfitting of the multivariate model, thereby affecting the results of the multivariate analysis. Nevertheless, these observations are consistent with findings reported in the current literature [1,2,3].

### Study Limitations

This analysis is subject to some limitations, primarily related to its single-center, non-randomized and observational design. We included a limited number of selected patients in the study in whom ‘single-shot’ procedures were performed. Only selected clinical, echocardiographic and periprocedural data were analyzed, with an imbalance regarding selected parameters. There is no information on the detailed general anesthetic protocol, which varied according to the experience of the anesthesiologist. Device selection was nonrandom and operator-dependent. Neither operator experience nor the learning curve was analyzed. Only the duration of the skin-to-skin procedure was analyzed, whereas the time required to prepare the patient for anesthesia was not assessed. Ultrasonography was not used to guide vascular access, which may have affected the incidence of complications in the compared groups. A small number of arrhythmia recurrences were observed, which may have been due to periodic rhythm monitoring.

## 5. Conclusions

In our single-center observational comparison of PVI using CBA, RFB, or PFA, procedures based on PFA resulted in shorter procedure times, primarily by reducing left atrium dwell time. No differences in the risk of arrhythmia recurrence were observed within 12 months after the procedure according to the “single-shot” ablation device used.

## Figures and Tables

**Figure 1:**
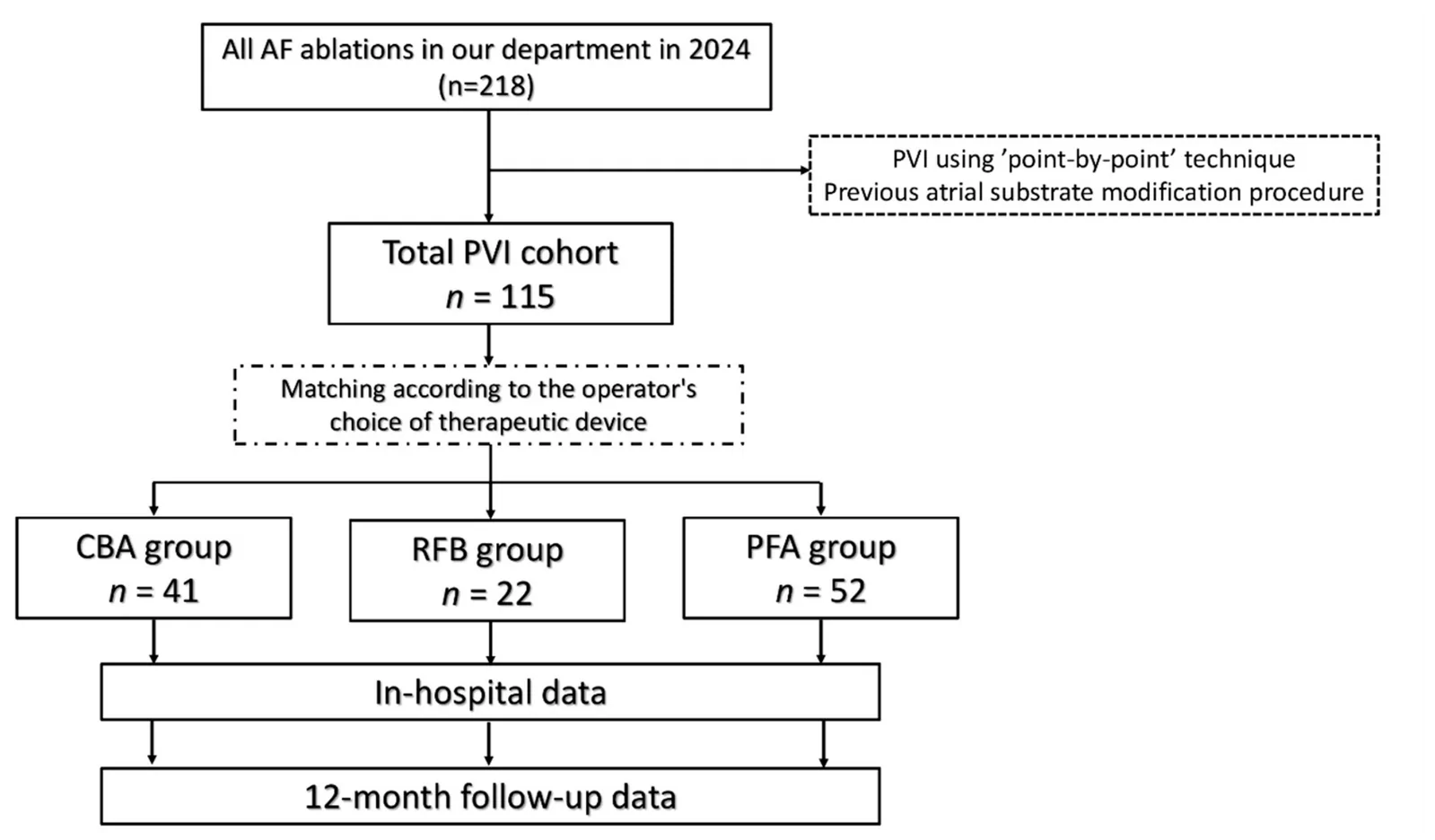
Flow diagram. The flow chart showing the selection process for the 115 patients included in the final analysis.

**Figure 2:**
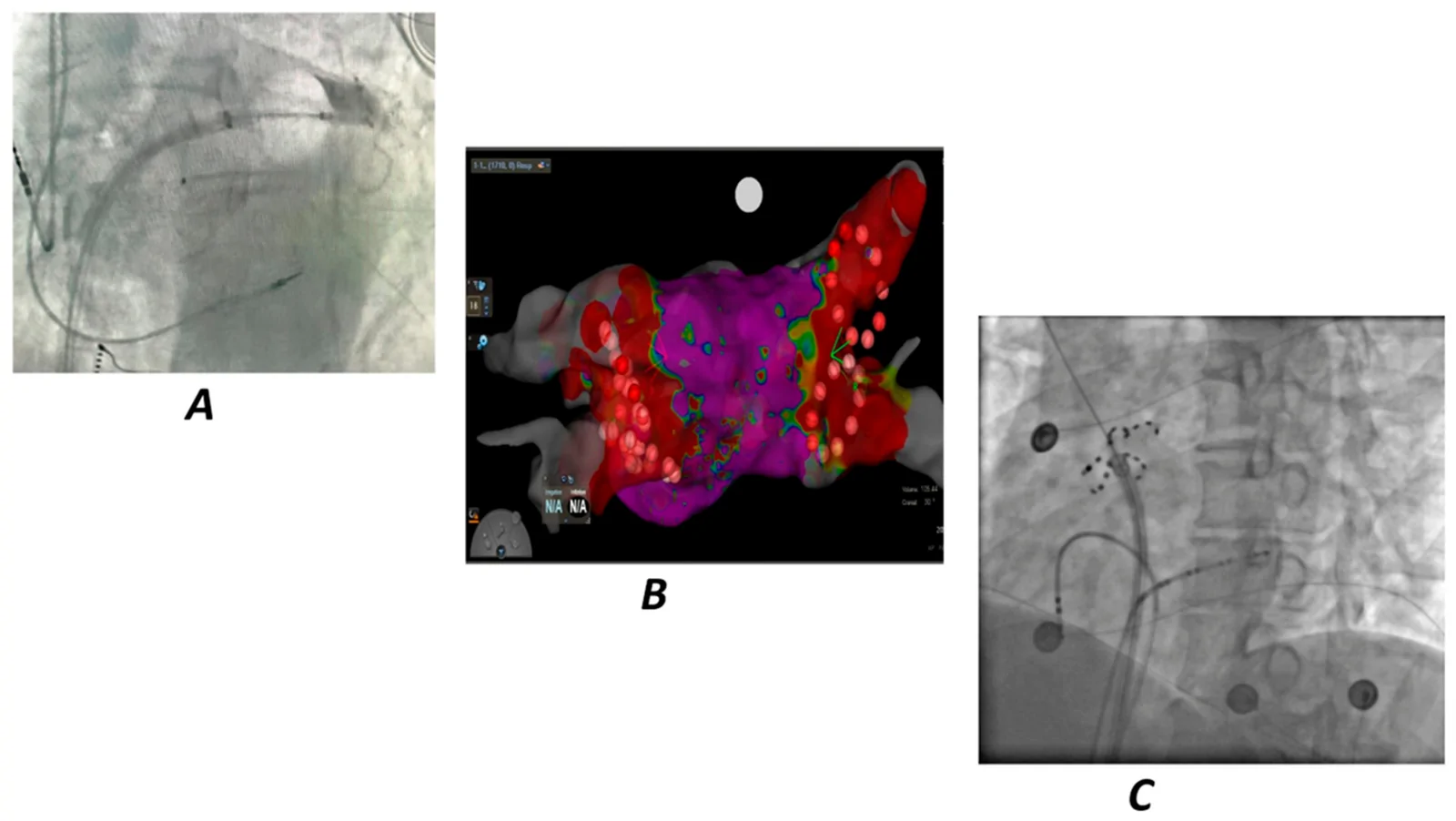
The figure illustrates various “single-shot” systems used to achieve pulmonary vein isolation in the patients included in this analysis. They are presented in the following order: (**A**) a second-generation cryoballoon positioned in the left superior pulmonary vein (AFA, Medtronic, Minneapolis, MN, USA); (**B**) a 3D electro-anatomical map created using a radiofrequency balloon, along with the corresponding ablation points (Heliostar, Biosense Webster, California, CA, USA); and (**C**) a pulsed field ablation system in a “flower” configuration, positioned in the right superior pulmonary vein (Farawave, Boston Scientific, Massachusetts, MA, USA).

**Figure 3:**
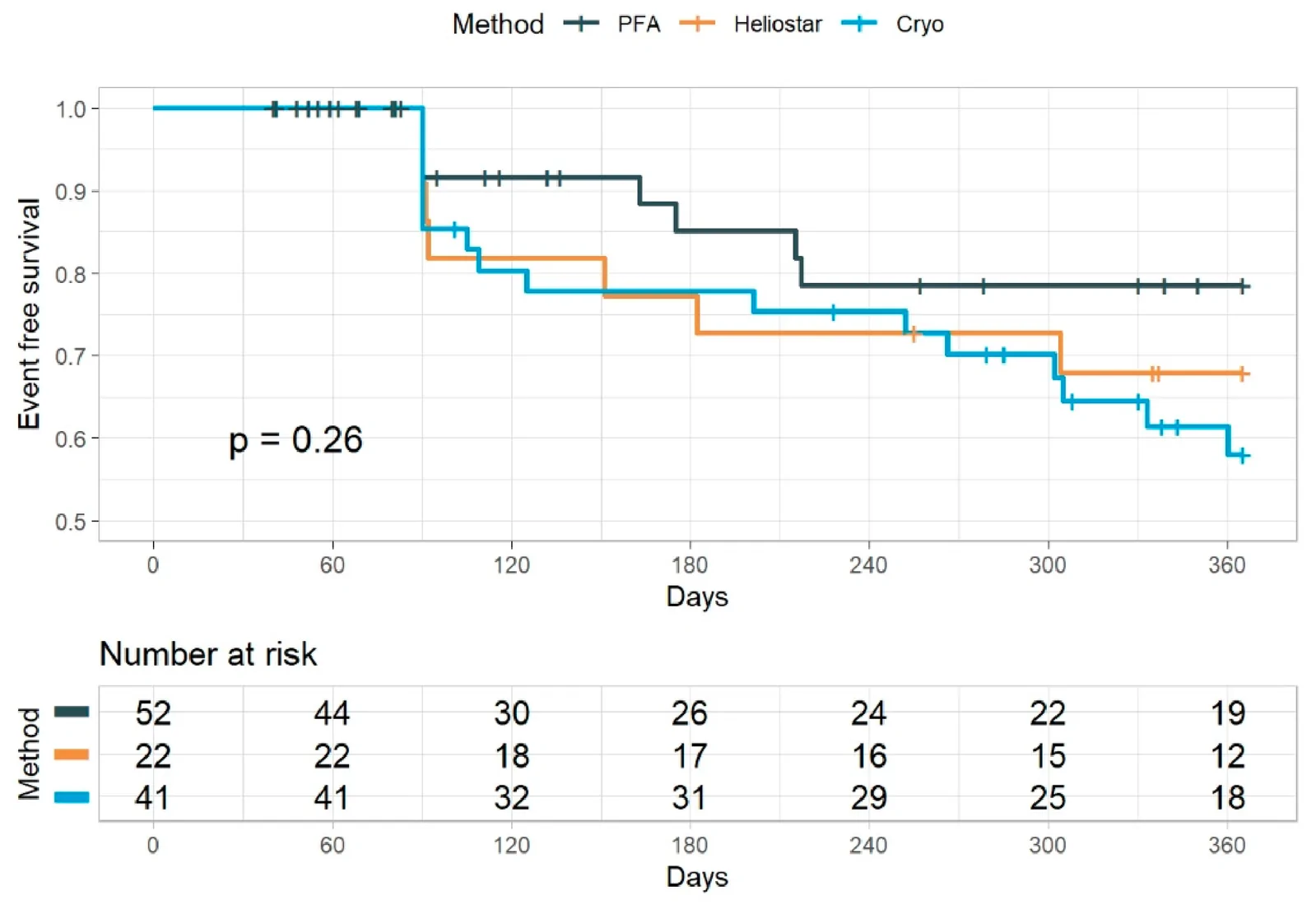
The Kaplan–Meier curves for arrhythmia-free survival according to the device used. The figure presents Kaplan–Meier curves for arrhythmia-free survival according to the different “single-shot” device used. The black curve represents procedures using PFA technology, the blue curve represents cryoablation procedures, and the orange curve represents procedures using a radiofrequency balloon. The figure includes the *p*-value from the log-rank test.

**Table 1:** Baseline data.

	Total (*n* = 115)	Cryo (*n* = 41)	RF Baloon (*n* = 22)	PFA (*n* = 52)	*p* Value
Age, years, median, IQR	66 (54, 73)	67 (58; 75)	63.5 (53; 70)	64 (54.5; 70.5)	0.211
Male sex, *n* (%)	73 (63.5)	25 (61.0)	13 (59.1)	35 (67.3)	0.733
BMI, kg/m^2^	30.2 ± 5.4	29.9 ± 5.8	30.6 ± 5.3	30.0 ± 5.1	0.866
Persistent AF, *n* (%)	49 (42.6)	17 (41.5)	9 (40.9)	23 (44.2)	0.949
CHA_2_DS_2_-VASc score, median, IQR	3.0 (1.0, 4.0)	3.0 (2.0, 4.0)	3.0 (1.0, 4.0)	2.5 (1.0, 4.0)	0.753
HAS-BLED score, median, IQR	1.0 (0.0, 2.0)	1.0 (0.0, 2.0)	1.0 (0.0, 2.0)	1.0 (0.0, 1.0)	0.800
Previous AF ablation, *n* (%)	12 (10.4)	0 (0.0)	1 (4.5)	11 (21.2)	0.001
Hypertension, *n* (%)	82 (71.3)	30 (73.2)	15 (68.2)	37 (71.2)	0.916
Diabetes mellitus, *n* (%)	35 (30.4)	11 (26.8)	7 (31.8)	17 (32.7)	0.820
CAD, *n* (%)	39 (33.9)	12 (29.3)	6 (27.3)	21 (40.4)	0.407
Heart failure, *n* (%)	57 (49.6)	19 (46.3)	10 (45.5)	28 (53.8)	0.705
Stroke/TIA, *n* (%)	4 (3.5)	1 (2.4)	2 (9.1)	1 (1.9)	0.323
Implanted cardiac device (PM/ICD), *n* (%)	8 (7.0)	2 (4.9)	3 (13.6)	3 (5.8)	0.499
LVEF, %, median, IQR	55 (48, 55)	55 (48, 55)	55 (48, 55)	53 (44.5, 55)	0.412
Left atrial diameter, mm	44 ± 6	44 ± 6	42 ± 7	44 ± 7	0.479
History of CKD, stages III–V, *n* (%)	16 (13.9)	7 (17.1)	3 (13.6)	6 (11.5)	0.779

AF, atrial fibrillation; BMI, body mass index; CAD, coronary artery disease; CKD, chronic kidney disease; ICD, implantable cardioverter-defibrillator; LA, left atrium; LVEF, left ventricular ejection fraction; PFA, pulsed field ablation; PM, pacemaker; RF, radiofrequency; and TIA, transient ischemic attack. Data are presented as mean ± SD or median (Q1–Q3) depending on the distribution and as numbers and percentages. The Kruskal–Wallis rank sum test, Wilcoxon rank sum test, Fisher’s exact test or Welch’s two sample *t*-test were used.

**Table 2:** In-hospital and periprocedural data.

	Total (*n* = 115)	Cryo (*n* = 41)	RF Baloon (*n* = 22)	PFA (*n* = 52)	*p* Value
In-hospital data					
Creatinine level on admission, µmol/L, median, IQR	84.0 (78.0, 98.0)	84.0 (78.0, 102.0)	82.5 (80.0, 89.0)	87.5 (77.0, 97.0)	0.807
Creatinine level at discharge, µmol/L, median, IQR	84.0 (74.0, 95.0)	88.0 (77.0, 96.0)	84.0 (71.0, 93.0)	81.0 (74.0, 92.5)	0.260
Skin-to-skin procedure time, minutes, median, IQR	75 (50, 100)	90 (80, 105)	82.5 (60, 100)	55 (45, 77.5)	<0.001
General anesthesia, *n* (%)	71 (61.7)	8 (36.4)	11 (26.8)	52 (100.0)	<0.001
Total fluoroscopy time, minutes, median, IQR	14.1 (10.3, 17.9)	14.7 (10.8, 20.6)	14.6 (9.8, 17.2)	13.9 (10.3, 16.9)	0.553
Fluoroscopy time after TSP, minutes, median, IQR	8.8 (6.3, 12.2)	9.0 (6.7, 13.3)	8.1 (6.3, 9.5)	8.9 (6.1, 11.4)	0.420
Left atrial dwell time, minutes, median, IQR	47.0 (30.0, 64.0)	60.0 (48.0, 77.0)	60.0 (35.0, 65.0)	31.5 (26.0, 40.0)	<0.001
Fluoroscopy dose after TSP, mGy, median, IQR	95 (45, 217)	71 (34, 155)	158 (60, 265)	112 (50, 212)	0.214
Total number of applications, median, IQR	8 (5, 33)	4 (4; 6)	6 (5; 8)	34 (32; 34)	<0.001
Periprocedural complications					
Groin hematoma, *n* (%)	2 (1.7)	0 (0.0)	1 (4.5)	1 (1.9)	0.472
Transient phrenic nerve palsy, *n* (%)	2 (1.7)	2 (4.9)	0 (0.0)	0 (0.0)	0.160
Pericardial effusion, *n* (%)	5 (4.3)	1 (2.4)	4 (18.2)	0 (0.0)	0.002
Tamponade, *n* (%)	2 (1.7)	1 (2.4)	0 (0.0)	1 (1.9)	>0.999
Stroke, *n* (%)	1 (0.9)	0 (0.0)	1 (4.5)	0 (0.0)	0.191
Total complications rate, *n* (%)	12 (10.4)	4 (9.6)	6 (27.3)	2 (3.8)	0.015
Antiarrhythmic drugs at discharge					
Beta-blocker, *n* (%)	106 (92.2)	38 (92.7)	18 (81.8)	50 (96.2)	0.105
Propafenone, *n* (%)	55 (47.8)	18 (43.9)	12 (54.5)	25 (48.1)	0.722
Flecainide, *n* (%)	2 (1.7)	0 (0.0)	0 (0.0)	2 (3.8)	0.675
Sotalol, *n* (%)	3 (2.6)	3 (7.3)	0 (0.0)	0 (0.0)	0.088
Amiodarone, *n* (%)	9 (7.8)	4 (9.8)	0 (0.0)	5 (9.6)	0.385

PFA, pulsed field ablation; and RF, radiofrequency. Data are presented as mean ± SD or median (Q1–Q3) depending on the distribution and as numbers and percentages. The Kruskal–Wallis rank sum test, Wilcoxon rank sum test, Fisher’s exact test or Welch’s two sample *t*-test were used.

**Table 3:** Twelve-month follow-up data.

	Total (*n* = 115)	Cryo (*n* = 41)	RF Baloon (*n* = 22)	PFA (*n* = 52)	*p* Value
Documented recurrence of AF/AFl/AT, *n* (%)	30 (26.1)	16 (39.0)	7 (31.8)	7 (13.5)	0.016
Re-hospitalization due to AF/AFl/AT, *n* (%)	5 (4.3)	1 (2.4)	3 (13.6)	1 (1.9)	0.100
Repeat ablation for AF/AFl/AT, *n* (%)	3 (2.6)	2 (4.9)	1 (4.5)	0 (0.0)	0.209

AF, atrial fibrillation; AFl, atrial flutter; AT, atrial tachycardia. Data are presented as numbers and percentages. The Kruskal–Wallis rank sum test was used.

**Table 4:** Univariable Cox regression analysis.

Variables	Hazard Ratio (95% CI)	*p* Value
Age	1.010 (0.978–1.043)	0.500
Male sex	1.135 (0.540–2.385)	0.700
BMI	1.035 (0.973–1.102)	0.300
Persistent AF	2.265 (1.098–4.671)	0.027
CHA_2_DS_2_-VASc score	1.007 (0.811–1.250)	>0.900
HAS-BLED score	1.079 (0.778–1.496)	0.600
Previous AF ablation	0.385 (0.052–2.830)	0.300
Hypertension	0.993 (0.442–2.231)	>0.900
History of CKD, stages III–V	1.712 (0.697–4.201)	0.200
Diabetes mellitus	0.651 (0.266–1.593)	0.300
CAD	1.212 (0.567–2.593)	0.600
Heart failure	1.879 (0.893–3.955)	0.100
Creatinine level on admission	1.001 (0.982–1.020)	>0.900
Creatinine level at discharge	1.014 (0.999–1.029)	0.071
LVEF	0.945 (0.907–0.984)	0.006
LA diameter	1.080 (1.018–1.144)	0.010
Skin-to-skin procedure time	1.013 (0.998–1.028)	0.100
Total fluoroscopy time	1.025 (0.979–1.074)	0.300
Periprocedural general anesthesia	1.266 (0.609–2.631)	0.500
Fluoroscopy time after TSP	1.043 (0.974–1.117)	0.200
Left atrial dwell time	1.016 (0.999–1.033)	0.063
Single-shot ablation device		
-PFA	**---**	**---**
-RF balloon	1.612 (0.565–4.597)	0.400
-Cryoballoon	2.068 (0.850–5.027)	0.110
Beta-blocker at discharge	0.837 (0.254–2.759)	0.800
Propafenone at discharge	0.402 (0.184–0.879)	0.022

AF, atrial fibrillation; BMI, body mass index; CAD, coronary artery disease; CKD, chronic kidney disease; CI, confidence interval; LA, left atrium; LVEF, left ventricular ejection fraction; PFA, pulsed field ablation; PM, pacemaker; RF, radiofrequency; TIA, transient ischemic attack; and TSP, transseptal puncture.

**Table 5:** Multivariable Cox regression analysis.

Variables	Hazard Ratio (95% CI)	*p* Value
Left atrial diameter	1.082 (1.015–1.153)	0.015
Single-shot ablation device		
-PFA	**---**	**---**
-RF balloon	2.303 (0.770–6.886)	0.135
-Cryoballoon	2.260 (0.918–5.562)	0.076
Propafenone at discharge	0.437 (0.196–0.975)	0.022

CI, Confidence interval; PFA, pulse field ablation; and RF, radiofrequency.

## Data Availability

The original contributions presented in this study are included in the article. Further inquiries can be directed to the corresponding author.
